# Plasma brain natriuretic peptide levels are elevated in patients with cancer

**DOI:** 10.1371/journal.pone.0178607

**Published:** 2017-06-01

**Authors:** Sachiko Bando, Takeshi Soeki, Tomomi Matsuura, Takeshi Tobiume, Takayuki Ise, Kenya Kusunose, Koji Yamaguchi, Shusuke Yagi, Daiju Fukuda, Takashi Iwase, Hirotsugu Yamada, Tetsuzo Wakatsuki, Michio Shimabukuro, Naoki Muguruma, Tetsuji Takayama, Ichiro Kishimoto, Kenji Kangawa, Masataka Sata

**Affiliations:** 1 Department of Cardiovascular Medicine, Institute of Biomedical Sciences, Tokushima University Graduate School, Tokushima, Japan; 2 Department of Cardio-Diabetes Medicine, Institute of Biomedical Sciences, Tokushima University Graduate School, Tokushima, Japan; 3 Department of Gastroenterology and Oncology, Institute of Biomedical Sciences, Tokushima University Graduate School, Tokushima, Japan; 4 National Cerebral and Cardiovascular Center Research Institute, Suita, Osaka, Japan; Kurume University School of Medicine, JAPAN

## Abstract

**Background:**

Natriuretic peptides have been proposed as biomarkers of cardiovascular disease, especially heart failure. Brain natriuretic peptide (BNP) has also been shown to be upregulated at the transcriptional and translational levels by pro-inflammatory cytokines in cardiac myocytes. Although we often measure plasma BNP levels in cancer patients, it remains unknown whether cancer-related inflammation affects the plasma BNP levels. We investigated the relationship between the BNP and human cancers.

**Methods:**

We retrospectively studied 2,923 patients in whom the plasma BNP levels and serum C-reactive protein (CRP) were measured and echocardiography was performed. Patients with clinically evident heart failure (NYHA II or higher), heart disease requiring medical treatment or surgery, renal dysfunction, and inflammatory disease were excluded. There were 234 patients in the final analysis. Blood sampling was performed before surgery and chemotherapy. In addition, we evaluated the relationship between the inflammation and plasma BNP levels in mouse models of colon cancer.

**Results:**

Of the 234 patients, 80 were diagnosed with cancer. Both the plasma BNP and serum CRP levels were significantly higher in cancer patients than those without. There were no significant differences in the echocardiographic parameters. There was a significant positive correlation between the plasma BNP and serum CRP levels in cancer patients (r = 0.360, P<0.01) but not in those without. In cancer patients, only the CRP correlated with the BNP independent of the age, creatinine level, hypertension, and body mass index. In addition, in nude mice with subcutaneous colon cancer, the plasma BNP level was elevated compared with that in non-cancer mice, and there was a significant relationship between the plasma BNP and serum levels of the inflammatory markers.

**Conclusions:**

In cancer patients, as well as colon cancer model mice, the plasma BNP levels were elevated, possibly due to cancer-related inflammation. The effect of cancer on the BNP levels should be considered when using BNP as an indicator of heart failure in cancer patients.

## Introduction

The family of natriuretic peptides plays an important role in the regulation of cardiovascular homeostasis and extracellular fluid volume. The natriuretic peptide system consists of at least three structurally homologous peptides, including atrial natriuretic peptide (ANP), brain natriuretic peptide (BNP), and C-type natriuretic peptide (CNP) [1,2]. ANP is synthesized primarily by the atrial myocardium, whereas BNP is synthesized mainly by the ventricular myocardium in response to a volume overload and increased wall tension [3]. Several lines of evidence suggest that BNP is superior to the other cardiac biomarkers including ANP for the prognostication and risk stratification in patients with heart failure [4–6]. Therefore, BNP and N-terminal (NT)-proBNP have been used as biomarkers for the diagnosis, guiding therapy, and prognostication, and have been studied in clinical trials and studies [7].

Recently, natriuretic peptides have been reported to reduce the number of small cell and squamous cell lung cancer cells and inhibit progression of several cancers, such as pancreatic cancer, breast cancer, small cell lung cancer, and prostate adenocarcinomas, *in vivo* and *in vitro* [8]. The underlying mechanism is hypothesized to be the inhibition of the mitogen activated extracellular signal-regulated kinases (ERK) 1/2 and DNA synthesis, mediated in part by cyclic GMP [9]. In addition, endogenous ANP has been shown to be expressed in the nucleus and cytoplasm of the human pancreatic adenocarcinomas [10]. ANP is also localized to the endothelium of capillaries and fibroblasts within these tumors [10].

On the other hand, inflammatory conditions are usually present before a malignant change occurs, and an oncogenic change generates an inflammatory microenvironment that promotes the development of tumors [11]. BNP has been shown to be upregulated at the transcriptional and translational levels by pro-inflammatory cytokines in cardiac myocytes [12]. Thus, we hypothesized that the natriuretic peptide levels are increased in cancer patients as a response to cancer-related inflammation. BNP has been measured in many patients with cancer to rule out heart disease in our hospital. Therefore, in the present study, we retrospectively investigated the relationship between the BNP concentrations and human cancers. In addition, to confirm the hypothesis, we evaluated the relationship between the inflammation and plasma BNP levels in nude mice with subcutaneous colon cancer.

## Materials and methods

### Patient characteristics

We retrospectively studied 2,923 patients in whom the plasma BNP, serum C-reactive protein (CRP), and creatinine levels were measured and echocardiography was performed in our hospital. Patients with clinically evident heart failure (NYHA II or higher), heart disease requiring medical treatment or surgery, severe renal dysfunction (defined as a GFR 30 mL/min/1.73 m^2^), and inflammatory disease were excluded. Patients with cancer after treatments such as surgery, chemotherapy, or radiation, were also excluded. We included 234 patients in the final analysis. The study protocol was approved by the Institutional Review Board of Tokushima University Hospital. The patient records were anonymized and de-identified prior to the analysis.

Eighty patients were diagnosed with cancer. In 71 cancer patients without leukemia or a multiple myeloma or primary macroglobulinemia, the tumor staging was based on the TNM classification system of the American Joint Committee on Cancer staging criteria.

### Laboratory data

Blood sampling was performed within two weeks before the surgery or chemotherapy. We examined the plasma BNP, serum CRP, and serum creatinine levels when the patients had symptoms suggestive of heart failure. In addition, we also examined the plasma BNP levels in the same patients who could be followed up after surgery or a full course of chemotherapy (48 out of 80 patients with cancer). In these patients, the blood sampling was performed at least 1 month after the completion of the surgery or chemotherapy to exclude any effect of the surgery or chemotherapy. The sample for the BNP assay was transferred into tubes containing 1.5 mg/mL of 2Na-EDTA. Immediately after a rapid centrifugation at 3,000 rpm for 5 minutes, the plasma BNP concentration was determined using a commercially available immuno enzymometric assay (ST E test “TOSOH” II BNP; Tosoh, Tokyo, Japan). In addition, we also examined the plasma BNP levels in the same patients who could be followed up after the surgery or a full course of chemotherapy.

### Echocardiography

Transthoracic echocardiography was performed in all 234 patients before the surgery or chemotherapy. We used the echocardiographic data obtained one week before or after the BNP measurement. The left ventricular ejection fraction (LVEF) was measured using a modified Simpson’s biplane method. The left ventricular end-diastolic dimension (LVDd) was measured with M-mode in the parasternal long axis view. The left ventricular mass index (LVMI) was calculated using Devereux’s formula with the diastolic measurements of the left ventricular internal diameter (LVID), interventricular septal thickness (IVST), and posterior wall thickness (PWT): LVMI (g/m^2^) = (1.04 [(IVST + LVID + PWT)^3^ − LVID^3^] − 14 g) / body surface area. In addition, the motion of the mitral annulus was recorded in the apical four-chamber view and a 4–5 mm sample volume was positioned sequentially at the lateral and septal corners of the mitral annulus. The peak early (E´) and late (A´) diastolic mitral annular velocities were determined, as well as the E/e´ (the mitral inflow E velocity to tissue Doppler e´ ratio).

### Mouse models of colon cancer

Female BALB/cA nu/nu mice (age 5 weeks) were purchased from CLEA Japan, Inc. (Tokyo, Japan) and maintained in the animal housing facility at Tokushima University Graduate School. Subcutaneous tumors were induced by an injection of 107 LIM 1215 cells in the groin at the age of 6–7 weeks. They were euthanized by cervical dislocation after the transthoracic echocardiography (SonoScape S6V) and blood sampling were performed at 6 weeks when the tumor size reached approximated 10 mm in diameter (maximum tumor size was 12mm). Mice with the tumor size less than 7mm were excluded. Then, we examined the plasma BNP and serum CRP levels, interleuin (IL)-1β, IL-6, and tumor necrosis factor (TNF)-α by an enzyme-linked immunosorbent assay.

The health status of all mice was monitored by a sentinel program throughout the experiments. All mice were housed under a 12-hour light/dark cycle, with food and water available ad libitum. All experimental procedures conformed to the guidelines for animal experimentation of Tokushima University.

### Statistical analysis

Continuous variables are expressed as means ± standard deviation (SD). Categorical data are presented as absolute values and percentages. With regard to the patient characteristics, laboratory measurements, and echocardiographic parameters, comparisons between 2 groups were performed using a Student’s t-test and comparisons among 4 groups were evaluated by a one-way analysis of variance (ANOVA) followed by a Bonferroni correction. A multivariate logistic regression analysis was performed to identify any clinical factors and laboratory parameters associated with the plasma BNP levels. Differences were considered statistically significant for a p<0.05.

## Results

### Patient characteristics

The baseline characteristics of the 234 patients included in the present study are shown in Table 1. The study patients were divided into cancer (before the treatment) and non-cancer groups. The cancer group included 80 patients (35%) and the non-cancer group consisted of 154 patients (65%). There were no significant differences in the age (Cancer (-): 67.9±13.7 vs. Cancer (+): 72.4±11.9 years), male/female ratio (Cancer (-): 66/88 vs. Cancer (+): 33/47), creatinine level (Cancer (-): 0.85±0.36 vs. Cancer (+): 0.85±0.43 mg/dl), or body mass index (Cancer (-): 22.5±3.5 vs. Cancer (+): 22.1±3.9 kg/m^2^) between the two groups. The cancer group (n = 80) consisted of 12 patients (15%) with malignant lymphomas, 8 patients (10%) with colon cancer, 8 patients (10%) with prostate cancer, 7 patients (9%) with gastric cancer, 7 patients (9%) with breast cancer, 5 patients (7%) with lung cancer, 4 patients (5%) with liver cancer, 4 patients (5%) with pancreatic cancer, and 25 patients (31%) with other types of cancer. The main clinical diagnosis or symptoms in the non-cancer group (n = 154) were hypertension (n = 37), chest pain/dyspnea caused by non-cardiac diseases (n = 21), cerebrovascular disease (n = 16), preoperative screening (n = 16), mild valvular heart disease that did not need treatment (n = 11), diabetes mellitus (n = 10), arrhythmias that did not need treatment (n = 8), respiratory disease (n = 7), dyslipidemia (n = 5), aortic aneurysm (n = 4), peripheral vascular disease (n = 4), and others (n = 15).

**Table 1:** Patient characteristics.

	Cancer (-) (n = 154)	Cancer (+) (n = 80)
Age (years)	67.9±13.7	72.4±11.9
Male/Female	66/88	33/47
Creatinine (mg/dl)	0.85±0.36	0.85±0.43
Body mass index (kg/m^2^)	22.5±3.5	22.1±3.9

### BNP and CRP levels in patients with and without cancer

**Fig 1:**
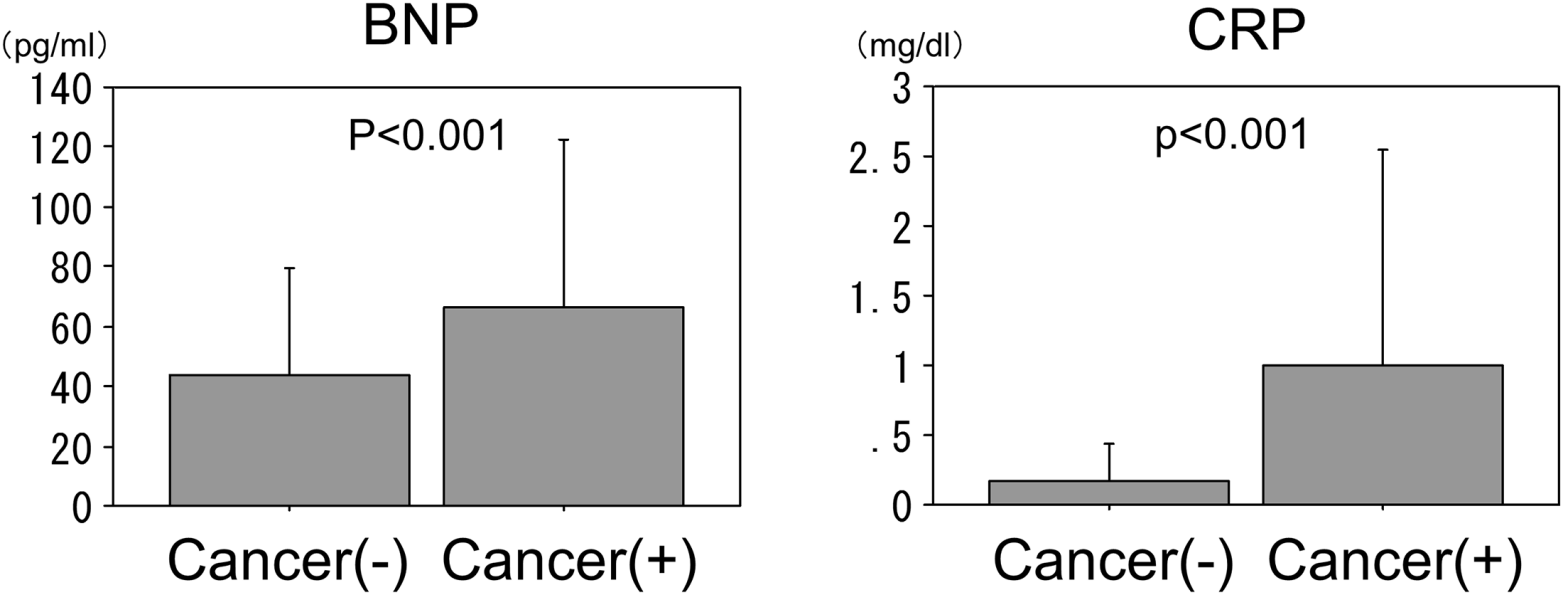
Plasma brain natriuretic peptide (BNP) and serum C-reactive protein (CRP) levels in patients with and without cancer. Both the BNP and CRP levels were significantly higher in the patients with cancer than in those without.

The plasma BNP levels were significantly higher in the patients with cancer than in those without (66.4±56.3 vs. 44.0±35.3 pg/ml, p<0.01). The serum CRP levels were also significantly higher in the patients with cancer than in those without (0.99±1.56 vs. 0.18±0.27 mg/dl, p<0.01) (Fig 1).

### BNP levels in the patients with different stages of cancer

**Fig 2:**
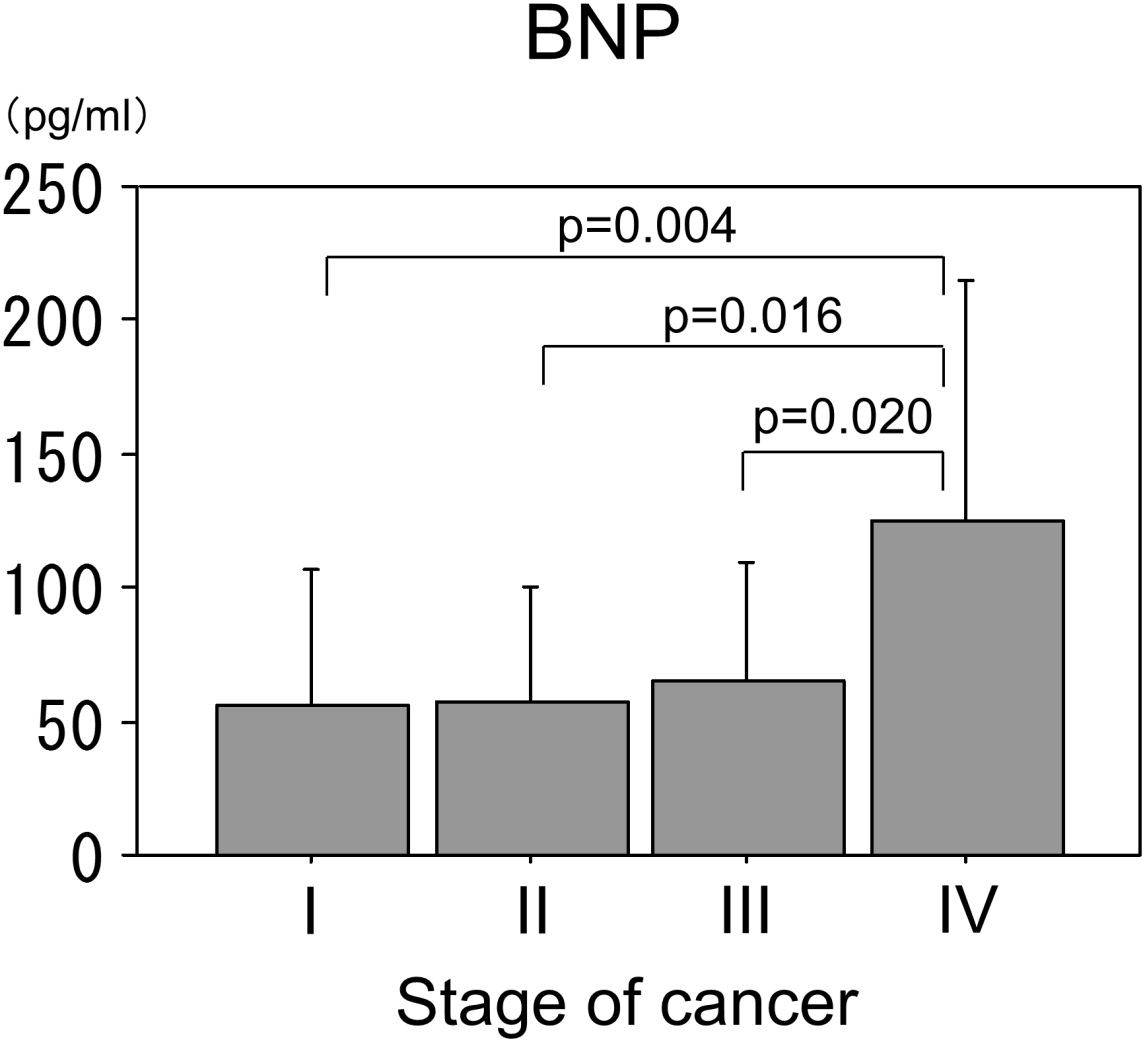
BNP levels in patients with different stages of cancer. Plasma BNP levels were significantly higher in cancer patients with stage IV than in patients with stage I, II, and III. There were no significant difference in BNP levels between cancer patients with stage I, II, and III. An ANOVA was used to assess the statistical significance followed by a Bonferroni correction.

The plasma BNP levels were significantly higher in the patients with stage IV cancer than in those with stage I, II, or III. There were no significant differences in the BNP levels between the cancer patients with stages I, II, and III (Fig 2).

### Echocardiographic parameters in the patients with and without cancer

The echocardiographic parameters of the LVEF (65.0±8.4 vs. 65.0±8.2%, p = 0.994), LVMI (112.0±37.0 vs. 118.3±38.3 g/m^2^, p = 0.231), LVDd (4.58±0.61 vs. 4.73±0.66 cm, p = 0.108), and E/e´ (9.4±3.9 vs. 10.1±3.7, p = 0.303) were similar in the patients with and without cancer. There were no echocardiographic findings suggestive of heart failure in the 234 patients with and without cancer.

### Relationship between the plasma BNP, serum CRP and creatinine levels, age, and body mass index

**Fig 3:**
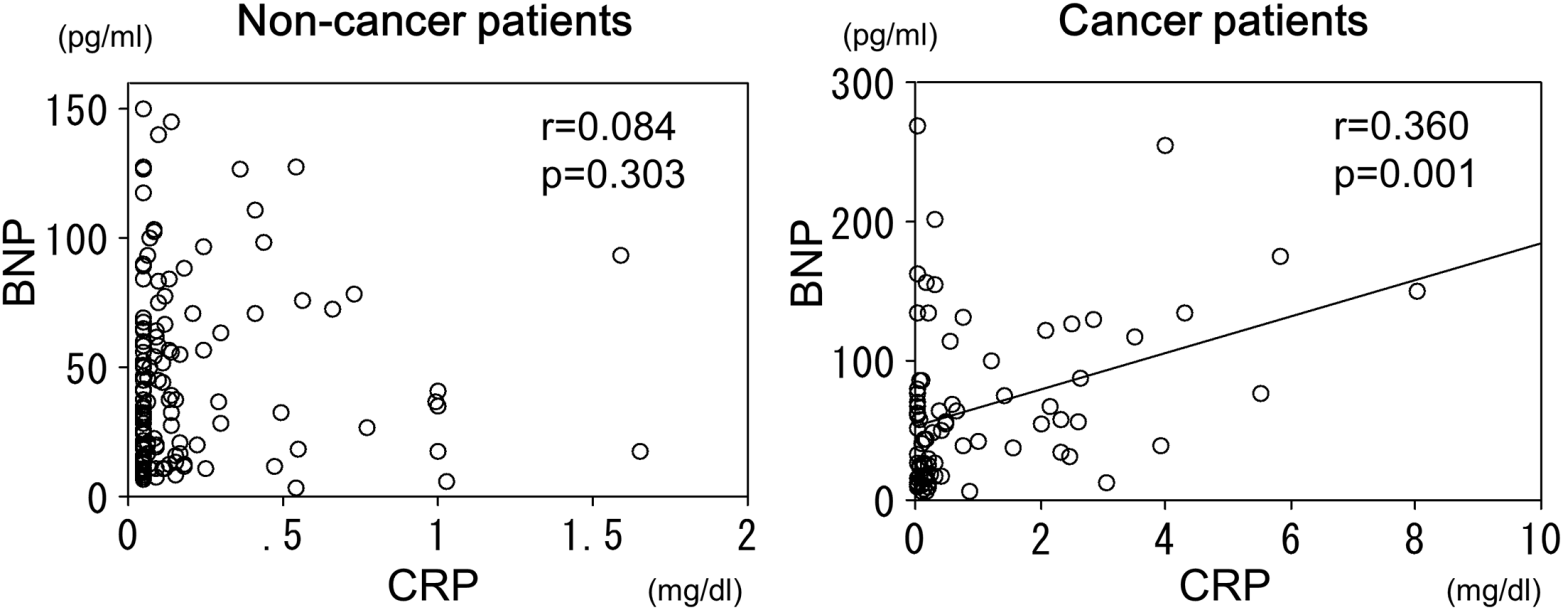
Relationship between BNP and CRP in patients with and without cancer. In non-cancer patients, there was no significant correlation between BNP and CRP levels, but there was a significant positive correlation between BNP and CRP levels in cancer patients.

**Fig 4:**
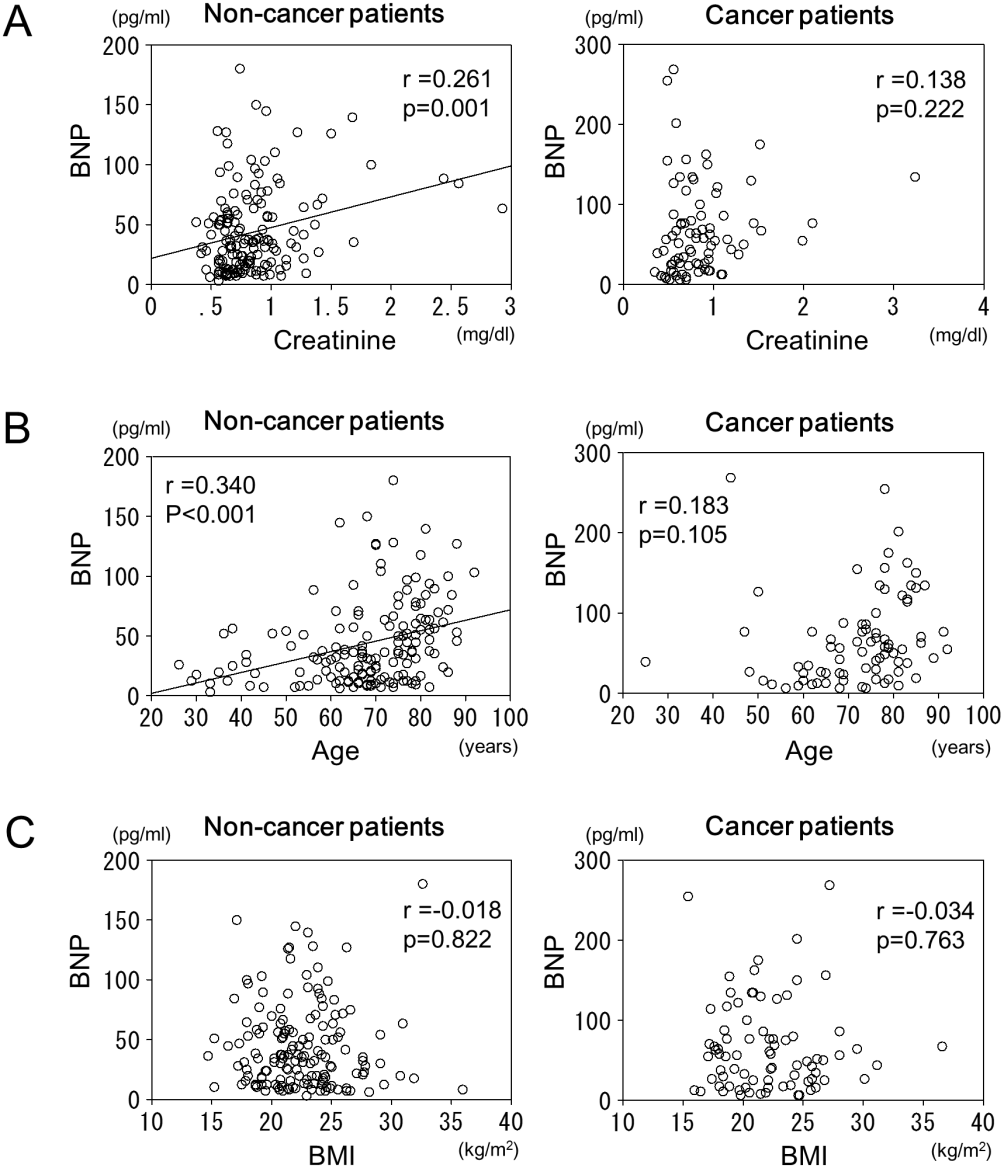
A. Relationship between the BNP and creatinine levels in the patients with and without cancer. In the non-cancer patients, there was a significant correlation between the BNP and creatinine levels, but there was no correlation between the BNP and creatinine levels in the cancer patients. B. Relationship between the BNP and age in the patients with and without cancer. In the non-cancer patients, there was a significant correlation between the BNP level and age, but that correlation was not observed in the cancer patients. C. Relationship between the BNP and body mass index in the patients with and without cancer. There was no significant correlation between the BNP levels and body mass index in either the patients with cancer or those without.

There was a significant positive correlation between the BNP and CRP levels in the cancer patients (r = 0.360, p<0.01), but not in the non-cancer patients (Fig 3). In the non-cancer patients, there was a significant positive correlation between the BNP and creatinine levels (r = 0.261, p<0.01), but not in the cancer patients (Fig 4A). There also was a significant positive correlation between the BNP levels and age in the non-cancer patients (r = 0.340, p<0.01), but not in the cancer patients (Fig 4B). There was no significant correlation between the BNP levels and body mass index in either the patients with cancer or those without (Fig 4C).

### Relationship between the plasma BNP and serum CRP levels in the patients with hematological cancers versus solid tumors

**Fig 5:**
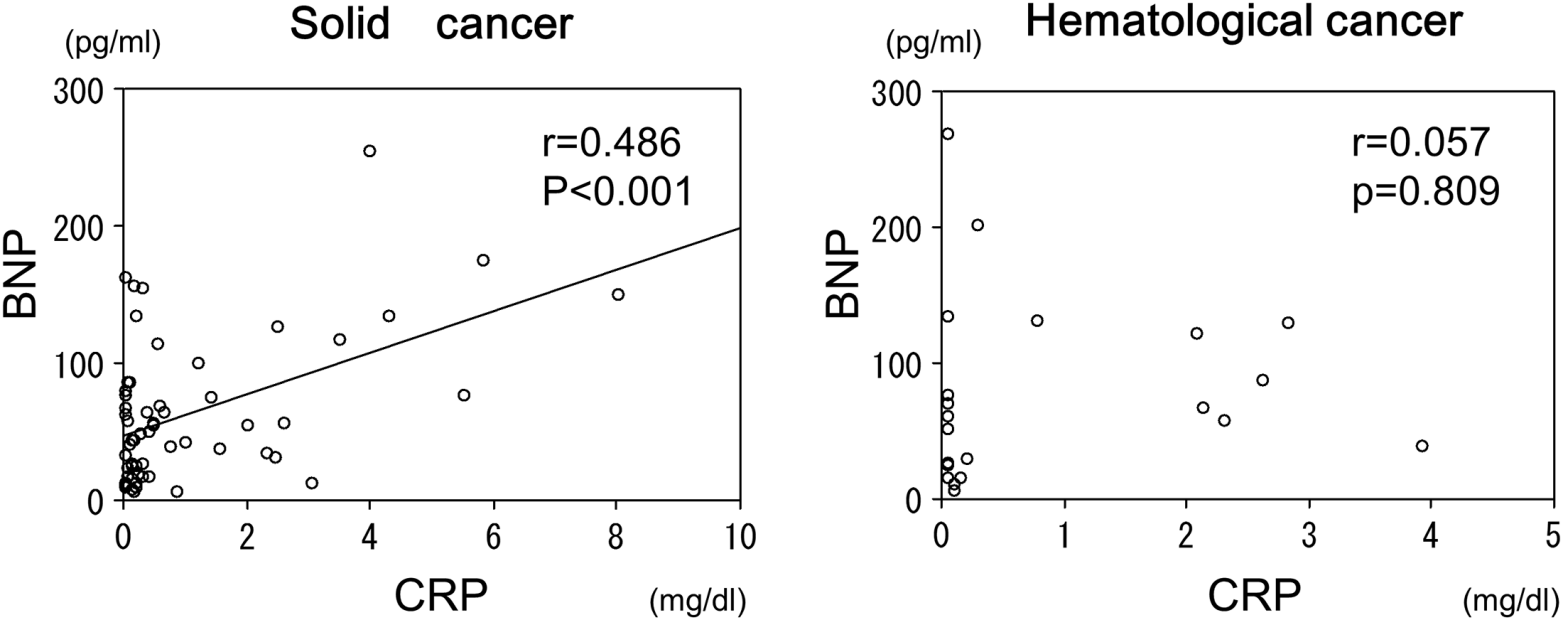
Relationship between the BNP and CRP levels in the patients with hematological cancers versus solid tumors. In the patients with hematological cancers, there was no relationship between the BNP and CRP levels. In contrast, there was a significant relationship between the BNP and CRP levels in the patients with solid tumors.

Since paraproteinemia associated with hematological malignancies may influence the BNP levels, we divided the cancer patients into those with hematological cancers (21 patients) and those with solid tumors (59 patients). In the patients with hematological cancers, there was no relationship between the BNP and CRP levels. In contrast, there was a significant positive relationship between the BNP and CRP levels in the patients with solid tumors (r = 0.486, p<0.01) (Fig 5).

### Multiple regression analysis of the BNP levels and potentially associated factors

A multiple regression analysis was performed to identify the factors associated with increased plasma BNP levels. In the patients with cancer, only the CRP concentration was associated with the BNP levels independent of the age, creatinine level, hypertension, body mass index, and the medication status (p<0.01) (Table 2). In the patients without cancer, the age and creatinine level were related to the BNP levels (Table 3).

**Table 2:** Multiple regression analysis of the BNP levels and potential associated factors in patients with cancer.

	Standard regression coefficient	t statistic	p value
CRP	0.361	3.088	0.003
Creatinine	-0.041	-0.302	0.764
Age	0.155	1.244	0.216
Hypertension	0.007	0.031	0.476
Body mass index	0.078	0.644	0.522
ACEI/ARB	-0.133	-0.673	0.503
β-blocker	-0.050	-0.399	0.691
Calcium antagonist	-0.012	-0.073	0.942
Diuretic	0.149	1.254	0.214
Statin	-0.209	-1.874	0.065

**Table 3:** Multiple regression analysis of the BNP levels and potential associated factors in patients without cancer.

	Standard regression coefficient	t statistic	p value
CRP	0.066	0.831	0.408
Creatinine	0.173	2.151	0.033
Age	0.246	2.957	0.004
Hypertension	-0.021	-0.141	0.888
Body mass index	-0.016	-0.201	0.841
ACEI/ARB	0.104	0.903	0.368
β-blocker	0.086	0.929	0.354
Calcium antagonist	0.076	0.740	0.461
Diuretic	0.090	1.123	0.263
Statin	0.005	0.060	0.952

CRP: C-reactive protein.

### Change in the plasma BNP levels before and after the surgery or chemotherapy

**Fig 6:**
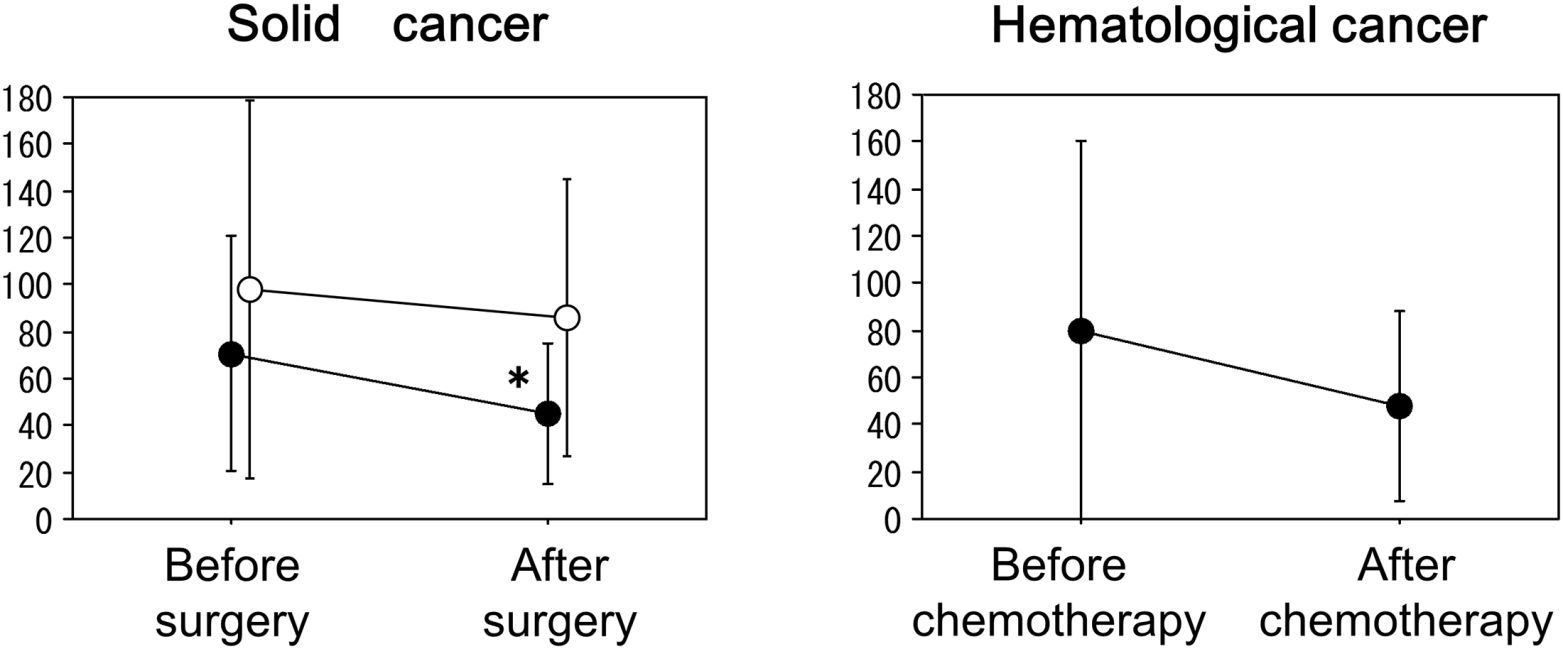
In 28 cured patients with solid cancers who underwent a radical surgery, the plasma BNP levels significantly decreased after the surgery (left panel, closed circles) (*p<0.01 vs the value before surgery). On the other hand, in 7 relapsed or insufficiently treated patients with solid cancers, the plasma BNP levels did not change after the surgery (left panel, open circles). In 13 patients with hematological cancers, the plasma BNP levels tended to decrease after the chemotherapy, but it did not reach a statistical significance.

In 28 cured patients with solid cancers who underwent a radical surgery, the plasma BNP levels significantly decreased from 70.7±49.9 pg/ml to 45.0±29.7 pg/ml after the surgery (p<0.01) (Fig 6). On the other hand, in 7 relapsed or insufficiently treated patients with solid cancers, the plasma BNP levels did not change after the surgery (from 98.8±80.7 pg/ml to 86.4±59.2 pg/ml, p = 0.290). In 13 patients with hematological cancers who could be followed up after chemotherapy, the plasma BNP levels did not change significantly after the chemotherapy (from 79.7±80.7 pg/ml to 47.7±40.7 pg/ml) (Fig 6). In addition, there was no significant relationship between the BNP and CRP levels after the surgery or chemotherapy in each group (data not shown).

### Relationship between the plasma BNP and serum levels of the inflammatory markers in mouse models of colon cancer

Compared with the control, mice with subcutaneous tumors with a size of approximated 10 mm induced by an injection of colon cancer cells (LIM 1215) had elevated plasma BNP levels (591.1±374.5 vs. 206.2±122.7 pg/ml). In addition, the serum levels of the CRP, IL-6, and TNF-α were significantly higher in the mice with colon cancer than in those without (CRP: 4.643±1.369 vs. 3.340±0.498 μg/ml, p<0.05; IL-6: 10.8±8.8 vs. 2.6±1.5 pg/ml, p<0.05; TNF-α: 0.48±0.15 vs. 0.28±0.06pg/ml, p<0.05), while the serum IL-1β did not change between the mice with and without cancer. In addition, the plasma BNP level was correlated with the serum levels of the CRP (r = 0.760, p<0.01), IL-6 (r = 0.600, p<0.05), and TNF-α (r = 0.625, p<0.05).

## Discussion

To the best of our knowledge, the present study was the first to demonstrate that there was a significant positive correlation between the plasma BNP and serum CRP levels in cancer patients as well as cancer model mice without overt heart failure. This finding suggested that the plasma BNP levels may have been elevated due to cancer-related inflammation in cancer patients. Therefore, we should carefully differentiate elevated BNP levels in cancer patients due to heart failure from cancer-related inflammation.

Elevated plasma BNP levels have been reported in patients with heart failure; BNP is used as a biomarker for the diagnosis and guiding therapy in patients with heart failure [7]. Therefore, in the present study, we excluded any patients with clinically evident heart failure and cardiac disease requiring medical treatment or surgery in order to eliminate the influence of heart failure on the BNP levels. In the present study, both the patients with and without cancer had a normal cardiac function. In addition to heart failure, the age, gender, obesity, hypertension, and renal function have been shown to substantially influence the BNP concentration [13–16]. In the present study, independent of possible associated factors, including the medication status, there were positive correlations between the BNP levels and the age or creatinine levels, respectively, in the non-cancer patients; this observation was consistent with the results of the previous studies. On the other hand, there were no significant correlations between the BNP levels and the age or creatinine levels, respectively, in the cancer patients. These findings might be due to the smaller number of cancer patients compared to non-cancer patients. Alternatively, cancer itself or cancer-related inflammation might influence the creatinine levels.

The plasma BNP levels were significantly higher in the patients with cancer than in those without with similar characteristics such as the age, gender, body mass index, blood pressure, renal function, and cardiac function as assessed by echocardiography. Furthermore, there was a significant positive correlation between the BNP and CRP levels in the cancer patients, which suggested that the plasma BNP levels may have been elevated due to cancer-related inflammation. In addition, the plasma BNP levels are increased in an advanced stage of cancer (stage IV), which might be accompanied by systemic inflammation. Furthermore, the plasma BNP levels significantly decreased after a radical surgery in the patients with solid cancers, and the plasma BNP levels tended to decrease after chemotherapy in the patients with hematological cancers.

For the above reasons, we should carefully determine whether the higher BNP levels are due to asymptomatic heart failure or the cancer itself in asymptomatic cancer patients. The weak correlationship between the BNP and CRP levels in cancer patients in the present study might suggest the inclusion of hidden heart failure. Although we excluded any patients with evident heart failure by careful echocardiography in the present study, we could not deny all possible heart failure because of the lack of any direct hemodynamic evidence. In addition, sarcopenia or skeletal muscle loss is a common problem in cancer patients and can negatively affect the physical function, which influences heart failure. In this kind of situation, an exercise tolerance test might be useful to detect any hidden heart failure, because patients with heart failure with a preserved ejection fraction display an impaired exercise capacity accompanied with an inadequate hemodynamic response during exercise [17].

Inflammatory conditions are present before a malignant change occurs, and an oncogenic change generates an inflammatory microenvironment that promotes the development of tumors [11]. Numerous endogenous molecules involved in cancer-related inflammation have been identified. These include transcription factors such as nuclear factor (NF)-κB and major inflammatory cytokines such as interleukin (IL)-1β, IL-6, and tumor necrosis factor (TNF)-α [18–21]. In particular, IL-6 is a key growth-promoting and anti-apoptotic inflammatory cytokine. Activated NF-κB is an effector of IL-6, which promotes neoplasia [21–23]. In addition, CRP is the prototypical short pentraxin. It is produced by the liver in response to IL-6 [24]. These findings suggest that, in the tumor microenvironment, inflammatory cells may produce cytokines, particularly IL-6, as a response to tumor cells, tissue necrosis, and associated inflammation. Furthermore, these cytokines may induce hepatocytes to synthesize CRP.

**Fig 7:**
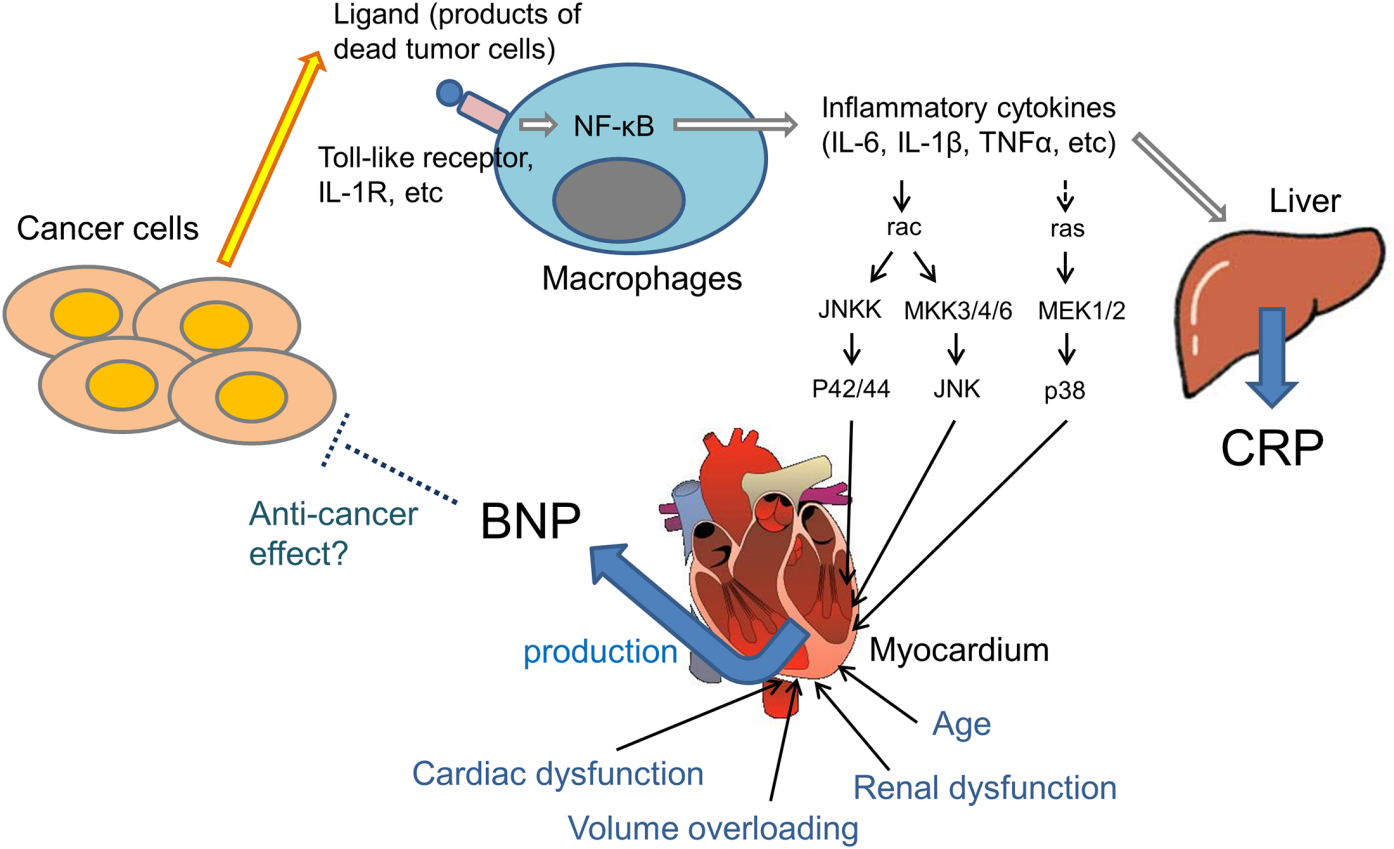
Hypothesized relationship between cancer and the BNP level. In the tumor microenvironment, inflammatory cells, including macrophages, produce various cytokines in response to tumor cells and tissue necrosis. In cardiomyocytes, BNP synthesis has been shown to be upregulated at the transcriptional level by inflammatory cytokines. Based on these previous findings and our present study, we hypothesized that the production of BNP might be increased in cancer patients by cancer-related inflammation independent of the cardiac function, renal function, and age.

BNP has been shown to be upregulated at the transcriptional and translational levels by pro-inflammatory cytokines in cardiac myocytes [12]. Pro-inflammatory signals are postulated to stimulate members of the mitogen-activated protein kinase (MAPK) family and c-Jun kinase, as well as other intracellular signaling cascades, which leads to the upregulation of the *BNP* gene expression. TNF-α and IL-1β induce a significant increase in the BNP mRNA and secretion via p38 MAPK in neonatal rat ventricular cardiomyocytes [25]. In addition, administration of lipopolysaccharide to healthy men increases the plasma NT-pro BNP levels without changing the heart rate or blood pressure [26]. In septic patients without systolic myocardial dysfunction, the plasma BNP is correlated with the CRP and IL-1β [27]. Furthermore, the plasma levels of NT-proBNP, CRP, and TNF-α in patients with rheumatoid arthritis (RA) without clinical heart failure are higher than in control groups, suggesting that increases in the NT-proBNP levels in RA patients are related to inflammation [28]. These findings support the notion that elevations in the plasma BNP occur in association with inflammation, independent of the cardiac function (Fig 7). This hypothesis was supported by our basic study showing that animal models with subcutaneous tumors induced by an injection of colon cancer cells (LIM 1215) had an elevated plasma BNP level compared with the control and that the plasma BNP level was correlated with the CRP, IL-6, and TNF-α serum levels.

In the present study, we also evaluated the correlation between the BNP and CRP levels in the patients with hematological cancers and solid tumors. There was a significant positive relationship between the BNP and CRP levels in the patients with solid tumors, but not hematological cancers. The measurement of the BNP might have been affected by paraproteinemia associated with hematological cancers. This finding is in agreement with a previous study showing that marked elevations in the BNP were more common in patients with solid tumors than hematologic malignancies [29]. This previous study also found no signs of a volume overload in more than 70% of cancer patients with a markedly elevated BNP. On the other hand, another report showed that patients with hematologic malignancies may have higher than expected NT-proBNP values in response to hypervolemic states, which may be related to a possible infiltration of the myocardium by substances produced in the setting of hematological cancers [30]. Further study is needed to clarify the effects of paraproteinemia on the BNP measurements.

The cardiac natriuretic peptides, particularly ANP, have been reported to inhibit the growth of cancers *in vitro* and *in vivo* [8,9]. The effects of cardiac natriuretic peptides on cancer may be mediated by intracellular messenger cyclic GMP formed by higher guanylate cyclase activity, which is part of the natriuretic peptide receptor-A complex. Cyclic GMP has been shown to have a strong anticancer effect, reducing human pancreatic cancer cell volume *in vivo* [9]. Natriuretic peptide receptor-A knockout mice do not express growth of implanted cancer cells [31]. Furthermore, a recent study has shown that ANP inhibits the adhesion of cancer cells to atrial and vascular endothelial cells by suppressing the E-selectin expression that is promoted by inflammation [32]. However, the present study was not designed to determine whether endogenously elevated plasma BNP levels have anticancer or antimetastatic effects in cancer patients.

To the best of our knowledge, there have been no previous reports showing that cancer cells generate BNP. Therefore, we think that an elevated plasma BNP level reflects the elevated production from cardiomyocytes in association with inflammation in cancer patients. However, a few studies reported an ectopic production of ANP mRNA in small cell lung cancer specimens from patients with this cancer [33,34]. Therefore, further studies are needed to evaluate the possible production of BNP from cancer itself.

Some limitations of the present study need to be acknowledged. First, although we showed the observational relationship between the BNP level and inflammation in cancer patients, the direct causative proof of the exact role of inflammation in increased BNP levels in patients with cancer is yet to be determined. Therefore, well-designed prospective studies to evaluate the relationship between the plasma BNP levels and cancer-related inflammation are needed in the future. Second, we could not confirm the absence of early-stage cancer by a systemic screening procedure in all “patients without cancer”. Instead, in the present study, we confirmed significantly decreased BNP levels after a radical surgery in solid-cancer patients who had undergone systemic screening examinations for other cancers and metastases. Further studies are needed to clarify the relationship between the BNP and CRP levels in “ab initio”-non-cancer patients validated by the systemic screening tests. Third, due to the small number and heterogeneity of the cancer patients, we could not evaluate the effect of the various origins or different stages of cancer on the results. Fourth, we could not provide an exact co-morbidity index, which could be a major contributor to the elevated inflammation, for all patients because of the unavailability of data due to the retrospective study design.

In conclusion, the plasma BNP levels were elevated in patients with cancer, probably due to cancer-related inflammation. This result suggested that we need to consider the effect of cancer on the BNP levels when we use the BNP as an indicator of heart failure in cancer patients. In other words, asymptomatic cancer patients with higher BNP levels should be diagnosed whether the elevated BNP is due to asymptomatic heart failure or cancer. Further studies to confirm these findings are needed.

## Supporting information

S1 Table This file contains the clinical data in human including BNP and CRP in this study. (XLSX)

S2 Table This file contains the basic data in mice including BNP and CRP in this study. (XLSX)
